# The role of SALL1-MGST1 axis-mediated ferroptosis inhibition in chemoresistance of retinoblastoma

**DOI:** 10.1371/journal.pone.0353000

**Published:** 2026-07-06

**Authors:** Zhuang Xiong, Weiwei Zhang, Xuebing Yu, Ruoshu Gu, Xue Zhang

**Affiliations:** Beijing Children’s Hospital, Heilongjiang Hospital, Harbin City, China; University of Hyderabad, INDIA

## Abstract

Retinoblastoma (RB) is the most prevalent intraocular malignant tumor in infants and young children, predominantly driven by the biallelic inactivation of the RB1 gene. Although multimodal treatment strategies have markedly improved the survival rates of pediatric patients, chemotherapy resistance, particularly carboplatin (CBP) resistance, remains a major clinical hurdle. In recent years, ferroptosis, an iron-dependent and lipid peroxide-driven form of programmed cell death, has garnered significant attention for its role in tumor drug resistance. This study investigates the functional mechanisms of the transcription factor Spalt-Like Transcription Factor 1 (SALL1) and its downstream target gene Microsomal Glutathione S-Transferase 1 (MGST1) in RB. Bioinformatics analysis revealed a significant upregulation of *SALL1* in RB, accompanied by enhanced activity of its regulatory network. Experimental evidence from ChIP-qPCR and luciferase reporter assays indicated that SALL1 binds to the promoter region of MGST1 and enhances its promoter transcriptional output. Functionally, knockdown of *SALL1* suppressed cell proliferation and induced ferroptosis, as evidenced by increased levels of lipid peroxidation, elevated malondialdehyde (MDA), and decreased glutathione (GSH) levels. These effects were reversible by the ferroptosis inhibitor Fer-1 or overexpression of MGST1. In the CBP-resistant cell line Y79-R, knockdown of *SALL1* notably reduced the IC50, enhanced chemosensitivity, and promoted cell death, a phenotype that could be rescued by overexpression of MGST1. Mechanistically, the SALL1-MGST1 axis promotes RB cell survival and drug resistance by inhibiting lipid peroxidation and ferroptosis. This study is the first to elucidate that the SALL1-MGST1 axis mediates CBP resistance in RB through the regulation of ferroptosis, providing a novel therapeutic target for reversing drug resistance.

## 1. Introduction

Retinoblastoma (RB) is the most prevalent intraocular malignant tumor in infants and young children. It originates from retinal photoreceptor precursor cells and accounts for 2–5% of pediatric malignancies. The global incidence is approximately 1 in 16,000–20,000 newborns, predominantly affecting children under 5 years old and constituting about 3% of all childhood cancers [1,2]. The development of RB is mainly attributed to the inactivation of both alleles of the tumor suppressor gene RB1, which leads to cell cycle dysregulation and tumor formation [3,4]. Common clinical manifestations include leukocoria, strabismus, and vision loss. In severe cases, it can lead to intraocular invasion, metastasis, and even death [5]. Treatment modalities mainly encompass local therapies (such as laser, cryotherapy, and plaque radiotherapy), chemotherapy, and enucleation [6]. Multimodal treatment has significantly improved survival rates [7], However, the prognosis depends on the timing of diagnosis, treatment regimen, and genetic factors. Early intervention can enhance survival and eye preservation, but lifelong follow-up is required to monitor the risk of secondary tumors [5].Therefore, in-depth research into the molecular mechanisms of RB, exploration of new therapeutic targets, and strategies to reverse drug resistance hold significant clinical importance.

With the advancement of molecular biology and genomics, studies have revealed that in addition to the RB1 gene, various alterations, including MYCN amplification, MDM2 overexpression, and epigenetic regulatory abnormalities, also play crucial roles in the development and progression of RB. These factors collectively promote tumor proliferation, invasion, and drug resistance [8]. Carboplatin (CBP), a first-line chemotherapeutic agent for RB, belongs to the alkylating agents and induces cell death by interfering with DNA repair [9,10]. However, long-term use of CBP often leads to drug resistance [11]. Hence, there is an urgent need to identify new therapeutic targets to enhance efficacy and overcome drug resistance. In recent years, ferroptosis, a novel form of iron-dependent programmed cell death characterized by the accumulation of lipid peroxides and an imbalance in antioxidant defense, has garnered considerable attention in tumor research [12,13]. Multiple studies have indicated that inducing ferroptosis may serve as a new strategy for treating drug-resistant tumors [14,15].

Spalt-Like Transcription Factor 1 (SALL1) is a zinc finger transcription factor that plays a critical regulatory role in embryonic development and tissue differentiation [16]. Recent research has shown that SALL1 exhibits abnormal expression in various tumors and may act as either an oncogene or a tumor suppressor gene, depending on the cancer type, thereby influencing tumor proliferation, differentiation, and apoptosis. For instance, in non-small cell lung cancer, high SALL1 expression is associated with a poor prognosis [17], whereas in renal cell carcinoma, downregulation of SALL1 expression exerts a tumor-suppressive effect [18]. The expression and function of SALL1 in RB remain unclear. Based on bioinformatic analysis, we hypothesize that SALL1 may participate in the progression of RB by activating the transcription of Microsomal glutathione S-transferase 1 (MGST1). MGST1 is a membrane-bound enzyme located in the endoplasmic reticulum and the outer mitochondrial membrane. It can catalyze the conjugation of glutathione with electrophilic substrates and possesses glutathione peroxidase activity, playing a key role in antioxidant and detoxification processes [19]. Studies have demonstrated that MGST1 is associated with chemotherapy resistance in various tumors and represents a potential therapeutic target. For example, in treatment-resistant melanoma, MGST1 is significantly upregulated, and its inhibition can disrupt redox homeostasis, suppress metastasis, and enhance the efficacy of chemotherapy and immunotherapy [20]. Additionally, MGST1 can impede the ferroptosis process by inhibiting lipid peroxidation [21].

In summary, despite the progress made in the treatment of RB, overcoming drug resistance and improving the prognosis of patients with advanced disease remain formidable challenges. The function of SALL1 in RB has not been fully elucidated, and the mechanism by which it may influence ferroptosis and drug resistance through the regulation of MGST1 warrants further exploration. This study aims to uncover the expression patterns, biological functions, and underlying molecular mechanisms of the SALL1-MGST1 axis in RB, with the intention of providing a new theoretical basis and experimental foundation for RB treatment strategies.

## 2. Materials and methods

### 2.1. Cell culture

The human RB cell line Y79 (HTB-18) was purchased from the American Type Culture Collection (ATCC). The CBP-resistant subline Y79-R was generated by continuous exposure of the parental Y79 cells to stepwise increasing concentrations of carboplatin (starting from 0.1 μM up to a maintenance concentration) over a period of 6 months, and was maintained under this selective pressure. Resistance was validated by a significantly elevated IC₅₀ for carboplatin compared to the parental line. All cell lines were authenticated by short tandem repeat (STR) profiling and confirmed to be free of mycoplasma contamination prior to use. The cells were routinely cultured in suspension using RPMI-1640 complete medium (12633020, ThermoFisher, USA; containing 10% fetal bovine serum, 100 U/mL penicillin, and 100 μg/mL streptomycin) in a saturated humidity incubator at 37°C with 5% CO₂. The cells were passaged at a 1:2 ratio every 2–3 days, and cells in the logarithmic growth phase were used for experiments.

### 2.2. Dataset collection and differential expression analysis

The normalized gene expression matrix of the GSE58780 [22] dataset was downloaded from the Gene Expression Omnibus (GEO) database (https://www.ncbi.nlm.nih.gov/geo/). Bioinformatics analysis of genes in the GSE58780 dataset was performed using the Hg-U133 Plus 2.0 Affymetrix gene array. Differentially expressed genes (DEGs) were identified using the “Limma” R package, with the criteria of |log₂ fold change (FC)| > 1.5 and false discovery rate (FDR) < 0.05 after Benjamini–Hochberg multiple testing correction. Volcano plots of the DEGs were generated using the “ggplot2” R package. To explore the biological functions of the genes, Gene Ontology (GO) and Kyoto Encyclopedia of Genes and Genomes (KEGG) analyses were performed using the “ClusterProfiler” package in R software, with a p-value threshold of < 0.05. The results were visualized using the “ggplot2” R package. Specifically, the DEGs between the RB group and the control group in the dataset were identified, and GO and KEGG analyses were conducted based on these DEGs.

### 2.3. Inference of *SALL1* regulon and Two-tail GSEA

Based on the R package RTN [23], we utilized gene expression data to construct a regulatory network that links *SALL1* to all its potential target genes. These target genes were referred to as the *SALL1* regulon. The network could be simplified or “filtered” by applying data processing inequality functions with increasingly stringent thresholds, thereby increasing the likelihood that the target genes assigned to *SALL1* were direct targets. Gene Set Enrichment Analysis (GSEA) was employed to assess the skewed distribution of a selected gene set within a gene list ranked according to a specific phenotype. In this study, GSEA was used to identify changes in the expression levels of the *SALL1* regulon induced by cancer. The two-tail GSEA method was based on the Connectivity Map procedure [24]. The regulon was divided into a positive target set (P) and a negative target set (N) based on the Pearson correlation coefficient. The gene phenotype was ranked using Log2(Foldchange). Subsequently, the distribution of P and N in the ranked phenotype was tested using GSEA statistics, generating independent enrichment scores (ES) for each subgroup. The differential enrichment score was calculated as dES = ESpos - ESneg. The two-tail GSEA analysis was implemented using the “tni,gsea2” function in the RTN R package (permutation = 1000).

### 2.4. Analysis of key genes related to immune cell infiltration and construction of functional networks

Based on published evidence of immune infiltration in RB, we applied the LM22 signature matrix to deconvolve immune cell proportions from ocular tumor transcriptomic data [25]. Functional networks were constructed using Cytoscape. The CIBERSORT algorithm was applied to analyze the microarray expression data of the samples, infer the relative proportions of 22 types of immune-infiltrating cells, and perform Pearson correlation analysis between *MGST1* expression and immune cell content. The deconvolution P‑values and root mean squared error (RMSE) for each immune cell type across all samples are provided in Supplementary Table 4. The “psych” R package was used to calculate the Pearson correlation between *MGST1* expression and the expression levels of other genes in the data, and the genes were ranked accordingly. Next, the fast gene set enrichment analysis (fgsea) algorithm in the “ClusterProfiler” R package was used to perform GSEA on the pre-ranked gene list. The number of permutations was set to 1000. A false discovery rate (FDR) < 0.05 was considered statistically significant for biological functions.

### 2.5. *SALL1* gene knockdown and overexpression

To reduce *SALL1* expression, specific siRNAs against *SALL1* (AM16708, Thermo Fisher Scientific, USA) and a negative control siRNA (4390843, Thermo Fisher Scientific, USA) were used. These siRNAs were transfected into Y79 cells at a final concentration of 50 nM using Lipofectamine 3000 transfection reagent (L3000001, Thermo Fisher Scientific, USA) when the cells reached 70–80% confluence. SALL1 expression levels were verified using qPCR and Western Blot.

A commercial lentiviral system was used to construct an *SALL1*-overexpressing Y79 cell model. The lentiviral vector carrying the human *SALL*1 gene (OE-SALL1) and the empty vector (EV) were purchased from Tsingke (China). Y79 cells in the logarithmic growth phase were seeded into 24-well plates at a density of 1 × 10⁵ cells per well. The viral solution was added at a multiplicity of infection (MOI) = 20, along with 8 μg/mL Polybrene. After 12 hours, the medium was replaced with fresh complete medium, and the cells were cultured for an additional 48 hours. Subsequently, the cells were cultured in medium containing 1.0 μg/mL puromycin for 7 days to select stable cell lines. SALL1 expression levels were verified using qPCR and Western Blot.

### 2.6. Real-Time Fluorescent Quantitative Reverse Transcription Polymerase Chain Reaction (RT-qPCR)

Total RNA was extracted from the cells using Trizol (15596026CN, Thermo Fisher Scientific, USA). The integrity, quantity, and purity of the RNA were examined using a spectrophotometer. The experimental system was prepared using the BeyoFast™ SYBR Green One-Step qRT-PCR Kit 171 (D7268M, Beyotime) and amplified using the SLAN Automatic Medical PCR Analyzer. The primer sequences are provided in Supplementary Table 1. The reaction conditions were set as follows: 5 minutes at 37°C, 10 minutes at 50°C, followed by 2 minutes at 94°C, and then 40 cycles of 15 seconds at 94°C and 30 seconds at 60°C. Data analysis was performed using the 2^−ΔΔCt^ method, and corresponding statistical analyses were conducted.

### 2.7. Western blot

The cells were lysed in RIPA lysis buffer (89901, Thermo Fisher Scientific, USA) containing protease inhibitors, followed by sonication to further promote cell lysis. The protein concentration was determined using a BCA protein assay kit (A55864, Thermo Fisher Scientific, USA). The protein samples were separated by SDS-PAGE and then transferred onto PVDF membranes. The membranes were blocked in 5% skim milk for 1 hour and then incubated overnight with primary antibodies at 4°C (SALL1, PA5–115864, Invitrogen, USA; MGST1, ab131059, Abcam, USA; GPX4, ab125066, abcam, USA; SLC7A11, ab307601, abcam, USA; β-actin, ab8227, Abcam, USA; diluted 1:1000). After washing three times with PBST (15 minutes each), the membranes were incubated with diluted secondary antibodies (Goat Anti-Rabbit IgG H&L (HRP), ab6721, Abcam, USA; diluted 1:1000) at room temperature for 1 hour. After washing four times with PBST, enhanced chemiluminescence was performed using ECL luminescent solution (P0018M, Beyotime, China). Density analysis of each band was performed using ImageJ software, and quantitative statistical analysis was conducted.

### 2.8. Chromatin immunoprecipitation-quantitative PCR (ChIP-qPCR)

To verify the direct binding of the transcription factor SALL1 to the promoter region of *MGST1*, a ChIP-qPCR experiment was conducted. Y79 cells were cross-linked and then sonicated to fragment the chromatin into 200–500 bp fragments. Immunoprecipitation was performed using a specific antibody against *SALL1* (PA5–115864, Invitrogen, USA), with normal IgG antibody serving as the negative control and the Input group as the positive control. The DNA fragments obtained from the precipitation were purified, and quantitative PCR was used to analyze specific fragments in the promoter region of *MGST1* where the predicted SALL1 binding sites were located (primer sequences are provided in Supplementary Table 2). The data were calculated and analyzed using the relative Input percentage method.The percentage of Input for the SALL1-bound MGST1 promoter fragment was approximately 2%, confirming specific enrichment. The experiment was independently repeated three times.

### 2.9. Luciferase reporter assay

To verify the transcriptional activation of the *MGST1* promoter by SALL1, a luciferase reporter system was used. The promoter sequence of *MGST1* was cloned into the pGL3-Basic vector to construct the reporter plasmid pGL3-*MGST1*-promoter. Y79 cells were seeded into 24-well plates at a density of 1 × 10⁵ cells per well and co-transfected with the reporter plasmid and pcDNA3.1-*SALL1* or the empty pcDNA3.1 vector using Lipofectamine 3000. After 48 hours of transfection, the cells were collected, and luciferase activity was measured using a dual-luciferase reporter gene assay kit (RG027, Beyotime, China). Renilla luciferase activity was used as an internal control for normalization. The experiment was independently repeated three times.

### 2.10. Cell Counting Kit-8 (CCK8) assay

Cells were seeded into 96-well plates, with approximately 1000–2000 cells and 100 μL of medium per well. After incubation in a 37°C incubator for 24 hours, 10 μL of CCK-8 reagent (96992, Sigma, USA) was added to each well. The optical density (OD) values of the cells were measured at 0, 24, 48, and 72 hours.

### 2.11. Colony formation assay

After corresponding transfection treatments, Y79 cells were seeded into 6-well plates at a low density (500 cells per well) and cultured in RPMI-1640 medium containing 10% fetal bovine serum for 14 days. The medium was refreshed every 3 days. At the end of the culture period, the medium was discarded, and the cells were fixed with 4% paraformaldehyde (441244, Sigma, USA) for 15 minutes and stained with 0.1% crystal violet for 30 minutes. The number of cell colonies with a diameter greater than 50 μm was counted, and the colony formation rate was compared among the groups. The experiment was independently repeated three times.

### 2.12. Lipid peroxidation assay

The lipid peroxidation level in Y79 cells was detected using a lipid peroxidation detection kit (BODIPY 581/591, C11S0043S, Beyotime, China) according to the manufacturer’s instructions. The reduced form of BODIPY 581/591 C11 has a maximum excitation wavelength of 581 nm and a maximum emission wavelength of 591 nm, predominantly emitting red fluorescence, resulting in a relatively large red-to-green fluorescence ratio. After oxidation by lipid hydroperoxides, the maximum excitation and emission wavelengths shift to approximately 488/510 nm, predominantly emitting green fluorescence, which reduces the red-to-green fluorescence ratio. The degree of lipid peroxidation can be determined based on this ratio. In this experiment, the positive control reagent LpoUp provided in the kit was not used.

### 2.13. Malondialdehyde (MDA) content assay

To evaluate the lipid peroxidation level in Y79 cells, the thiobarbituric acid (TBA) assay was used to detect the intracellular MDA content. Cells from different treatment groups were collected, washed with pre-cooled PBS, and lysed in cell lysis buffer on ice for 30 minutes. After centrifugation at 12,000 rpm for 10 minutes, the supernatant was collected. The MDA detection kit (BC0025, Solarbio, China) was used strictly according to the instructions: an appropriate amount of supernatant was mixed thoroughly with the TBA working solution, heated in a 95°C water bath for 40 minutes, cooled, and the absorbance was measured at 532 nm. The MDA concentration in each sample was calculated based on the standard curve and normalized to the total protein concentration. The experiment was independently repeated three times.

### 2.14. GSH content assay

To detect the intracellular GSH level in Y79 cells, a GSH and GSSG detection kit (S0053, Beyotime, China) was used. The following steps were briefly performed: approximately 1 × 10⁶ cells from different treatment groups were collected, washed with pre-cooled PBS, and mixed with the protein removal reagent provided in the kit. The mixture was vortexed on ice and allowed to stand for 10 minutes, followed by centrifugation at 10,000 rpm at 4°C for 10 minutes. The supernatant was collected as the sample to be tested. According to the kit instructions, the sample was mixed with the chromogenic agent and incubated at 37°C in the dark for 20 minutes, and the absorbance was measured at 420 nm. The absolute GSH concentration in the sample was calculated based on the standard curve and normalized to the total protein concentration determined by the BCA method.

### 2.15. IC50 assay

Cells in the logarithmic growth phase were seeded into 96-well plates at a density of 5 × 10³ cells per well. Blank zero-adjustment wells and different concentration groups of CBP (0–1000 μM, C2538, Sigma, USA) treatment groups were set up, with 5 replicate wells per group [26–28]. The cells were cultured at 37°C with 5% CO₂ for 48 hours. Then, 10 μL of CCK-8 solution was added to each well, and the cells were incubated for an additional 2 hours. The absorbance of each well was measured at 450 nm using a microplate reader. GraphPad Prism 9 was used to perform non-linear regression fitting, with the CBP as the x-axis (logarithmically transformed) and the cell survival rate of Y79 and Y79-R cells as the y-axis. The half‑maximal inhibitory concentration (IC₅₀) values, along with the Hill slope and 95% confidence intervals (95% CI), were derived from the fitted curves. The experiment was independently repeated three times.

### 2.16. Live/Dead cell staining assay

Cell viability and death were assessed using a Live/Dead Cell Double-Staining Kit (04511, Sigma, USA). Briefly, after treatment, cells were collected and washed twice with pre-warmed PBS. The cells were then resuspended in 1 mL of staining buffer containing 2 μM Calcein-AM and 4.5 μM Propidium Iodide (PI) and incubated at 37°C in the dark for 15–20 minutes. After incubation, the cells were washed once with PBS and immediately imaged under a fluorescence microscope. Live cells (with intact esterase activity) were stained green by Calcein-AM (excitation/emission: ~ 494/517 nm), while dead cells (with compromised plasma membrane integrity) were stained red by PI (excitation/emission: ~ 535/617 nm). At least three random fields per sample were captured, and the numbers of live (green) and dead (red) cells were counted using ImageJ software.

### 2.17. Statistical analysis

All statistical analyses of the experimental results were performed using GraphPad Prism 9. The sample size (n) for all experiments represents the number of independent biological replicates. The normality of data distribution was assessed using the Shapiro-Wilk test, and the homogeneity of variances was verified using Levene’s test. For data meeting the assumptions of normality and homogeneity of variances, parametric tests were applied (unpaired two-tailed t-test for two-group comparisons or one-way ANOVA with Tukey’s post hoc test for multiple comparisons). Effect sizes (Cohen’s d for t‑tests, partial η² for ANOVA) are reported where applicable. The data were presented as the mean ± standard deviation (mean ± SD). A p-value < 0.05 was considered statistically significant.

## 3. Results

### 3.1. Differential gene analysis reveals upregulation of *SALL1* in RB

**Fig 1:**
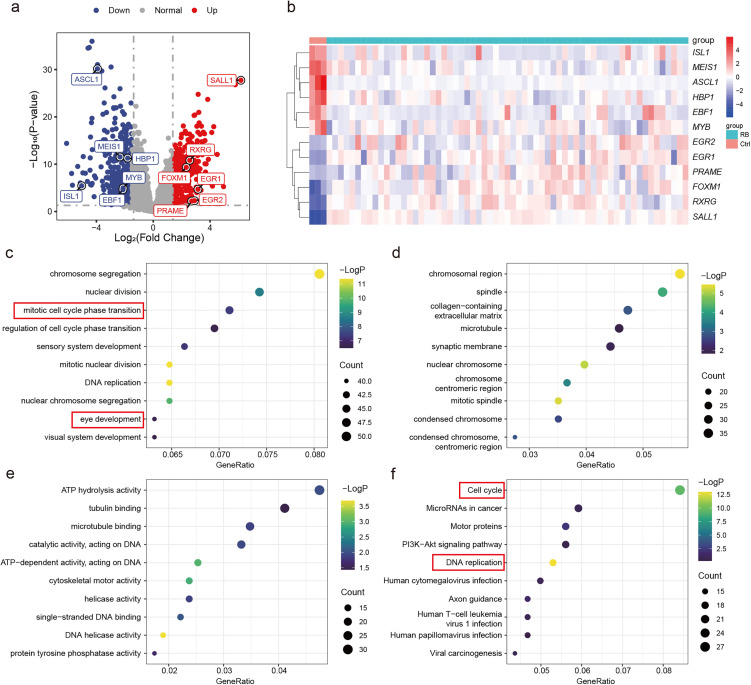
Differential Gene Analysis Reveals Upregulation of *SALL1* in RB. (a) Volcano plot showing the distribution of DEGs between the RB group and the control group. (b) Heatmap showing the distribution of DEGs between the RB group and the control group. (c-e) GO analysis of DEGs between the RB group and the control group. (f) KEGG analysis of DEGs between the RB group and the control group.

Differential analysis based on the GSE58780 dataset revealed 337 upregulated genes and 374 downregulated genes between the RB group and the control group (Fig 1a). The top 10 most significantly upregulated and downregulated genes were selected to generate a heatmap (Fig 1b). It was found that among the upregulated genes in RB tissues, the gene encoding the transcription factor SALL1 exhibited the most significant upregulation, demonstrating both the largest log2 fold change (logFC) and the most significant p-value. This expression profile suggests a positive correlation between *SALL1* and the disease progression of RB. The coordinated upregulation of *SALL1* along with genes such as *FOXM1* and *PRAME* may collectively promote malignant proliferation and immune evasion of tumor cells. In contrast, the significant downregulation of differentiation-related genes such as *MEIS1* and *ISL1* may indicate impaired cell differentiation and loss of retinal function. GO and KEGG enrichment analyses revealed that the DEGs were significantly enriched in multiple biological processes. In biological processes (BP) (Fig 1c), they were mainly enriched in “mitotic cell cycle phase transition,” indicating typical cell cycle checkpoint dysregulation and abnormal proliferation in RB. Additionally, the significant enrichment of the “eye development” process suggests a close relationship between tumor development and the reprogramming and differentiation blockade of retinal-specific developmental programs. In cellular components (CC), genes were enriched in mitotic structures such as the spindle and kinetochore (Fig 1d). In molecular functions (MF), they were primarily enriched in ATP-dependent DNA binding activity and microtubule binding activity (Fig 1e). KEGG analysis showed significant enrichment of DEGs in the cell cycle and DNA replication pathways, revealing the core mechanisms underlying uncontrolled proliferation and genomic instability in RB. The activation of the PI3K-Akt signaling pathway indicates significant survival advantages and apoptotic evasion in tumor cells (Fig 1f). These results collectively suggest the presence of cell cycle dysregulation, genomic instability, and an abnormal proliferation-differentiation balance in RB, providing a basis for core mechanism research and targeted therapy.

### 3.2. Construction of the *SALL1* regulatory network and functional analysis in RB

**Fig 2:**
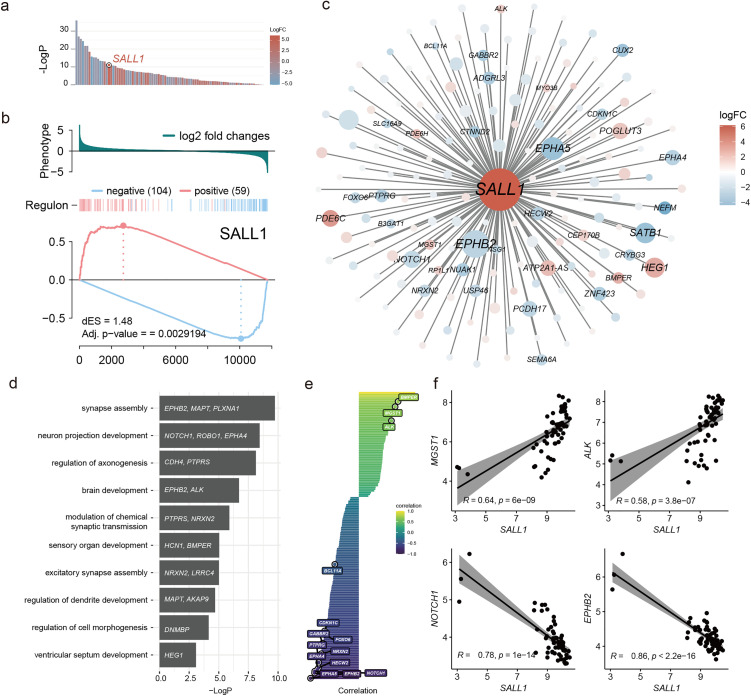
Construction of the *SALL1* Regulatory Network and Functional Analysis in RB. (a) Significance of transcription factor (TF) activity changes, with colors representing the fold change in TF expression between groups. (b) Double-tailed GSEA analysis of SALL1. (c) Regulatory network of *SALL1*. (d) Enriched pathways of *SALL1* target genes. (e-f) Bar charts and scatter plots showing the correlation between *SALL1* scores and target gene scores.

To elucidate the regulatory role of the transcription factor *SALL1* in RB, this study constructed an *SALL1*-centered transcriptional regulatory network (*SALL1* regulon) using the R package RTN based on gene expression data. The network inferred target gene relationships through mutual information algorithms and validated their statistical significance using permutation tests and bootstrap methods (Fig 2a–c). The analysis revealed highly significant changes in *SALL1* regulatory activity, with the regulon comprising 59 positively regulated targets and 104 negatively regulated targets. In RB tissues, two-tailed GSEA analysis indicated significant changes in *SALL1* regulon activity (dES = 1.48, p = 0.0029). Functional enrichment analysis of the *SALL1* regulon using ClusterProfiler revealed significant enrichment in pathways such as “sensory organ development” and “excitatory synapse assembly” (Fig 2d). The former suggests a critical role of *SALL1* in the differentiation and maintenance of retinal photoreceptor cells, with its dysfunction potentially closely related to the cellular origin of RB. The latter may affect inter-neuronal signal transmission and participate in the abnormal behavior of tumor cells. Further sample expression correlation analysis validated the regulatory relationship between *SALL1* and its target genes (Fig 2e–f). Among them, *MGST1* showed a significant positive correlation with *SALL1* expression, indicating that *MGST1* is a direct downstream target of *SALL1*. *MGST1*, a membrane-bound enzyme localized in the endoplasmic reticulum and mitochondrial outer membrane, reduces phospholipid hydroperoxides through its glutathione peroxidase activity, inhibiting lipid peroxidation. It is a key inhibitor of ferroptosis and a potential therapeutic target for cancer [29]. These findings suggest that *SALL1* may enhance the antioxidant and detoxification capabilities of tumor cells by upregulating *MGST1*, accelerate drug efflux from tumor cells, leading to drug inactivation and resistance. Additionally, *SALL1* may reduce tumor cell sensitivity to ferroptosis by upregulating *MGST1*, thereby decreasing tumor cell death.

### 3.3. Immune microenvironment regulation and functional enrichment analysis of MGST1

**Fig 3:**
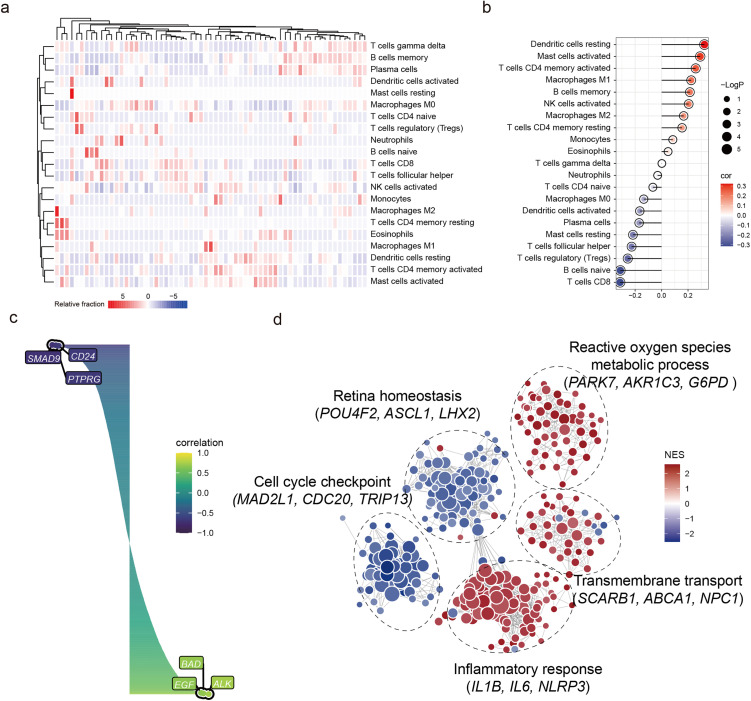
Immune Microenvironment Regulation and Functional Enrichment Analysis of *MGST1.* (a) Heatmap showing the distribution of immune cell proportions. (b) Lollipop plot showing the correlation between *MGST1* expression levels and immune cell content. (c) Bar chart showing the correlation between *MGST1* scores and other gene scores. (d) Biological function network influenced by *MGST1*, as determined by GSEA.

This study used the CIBERSORT algorithm to calculate the infiltration proportions of 22 immune cell types in the samples (Fig 3a). Correlation analysis revealed that MGST1 expression was significantly positively associated with resting dendritic cells and activated mast cells, was significantly negatively associated with Regulatory T Cells (Tregs), naive B cells, and CD8^+^ T cells (Fig 3b). This suggests that high MGST1 expression may be associated with the formation of an immunosuppressive microenvironment and weakened anti-tumor immune responses. To further explore the function of *MGST1*, we analyzed its correlation with other genes (Fig 3c). *MGST1* showed a significant positive correlation with the pro-apoptotic gene *BAD*, the transcriptional repressor *ERF*, and the receptor tyrosine kinase *ALK*. Conversely, *MGST1* exhibited a significant negative correlation with SMAD9, a key regulator of the BMP signaling pathway; *CD24*, an immune-regulatory membrane protein; and *PTPRG*, a tumor suppressor-associated tyrosine phosphatase. These results suggest that *MGST1* may be involved in regulating various biological processes, including cell apoptosis, signal transduction, and the immune microenvironment. GSEA enrichment analysis based on genome-wide Spearman correlations (MsigDB C5 GO BP database) revealed a significant positive correlation between *MGST1* expression and the “reactive oxygen species metabolic process” (Fig 3d), indicating that *MGST1* may play a central role in regulating intracellular redox balance. In RB, high *MGST1* expression may enhance cellular metabolic capacity for reactive oxygen species, reduce oxidative stress damage, and thereby inhibit oxidative stress-related cell death pathways such as ferroptosis, which might ultimately promot tumor cell survival and the formation of chemotherapy resistance.

### 3.4. SALL1 binds to the MGST1 promoter and enhances its activity in RB

**Fig 4:**
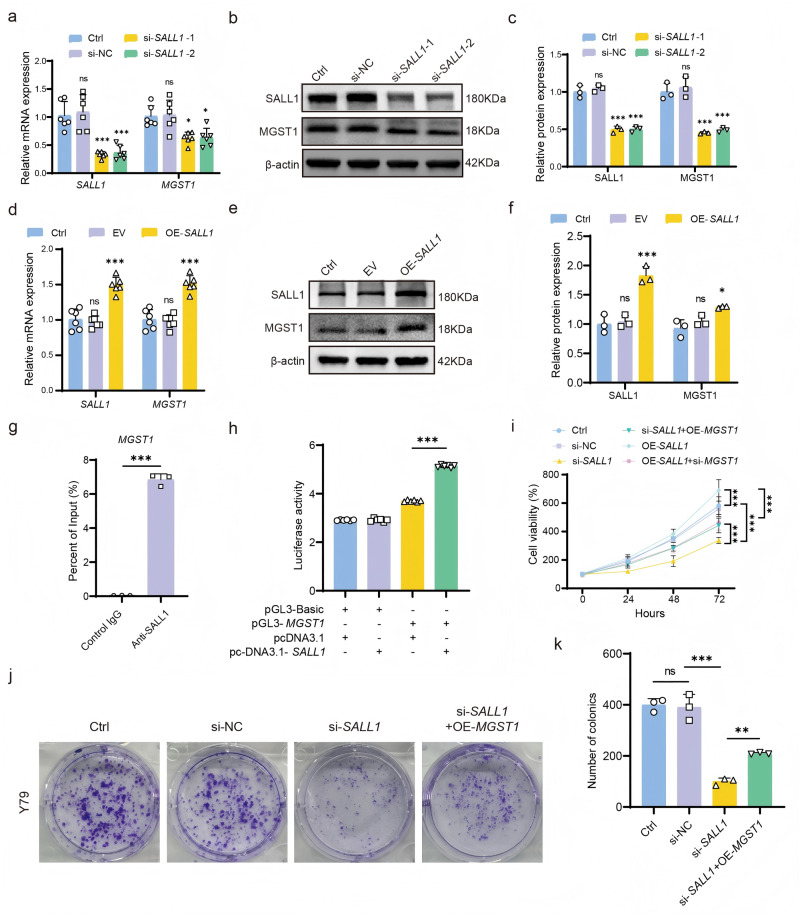
SALL1 Binds to the MGST1 Promoter and Enhances its Activity in RB. (a) qPCR results showing that *SALL1* knockdown downregulates *MGST1* mRNA expression (n = 6). *MGST1*, Ctrl vs si-*SALL1*–1, p = 0.01; Ctrl vs si-*SALL1*–2, p = 0.01. (b-c) Western blot results showing that *SALL1* knockdown downregulates MGST1 protein expression (n = 3). MGST1, Ctrl vs OE-*SALL1*, p = 0.01. (d) qPCR results showing that *SALL1* overexpression upregulates *MGST1* mRNA expression (n = 6). (e-f) Western blot results showing that *SALL1* overexpression upregulates MGST1 protein expression (n = 3). (g) ChIP-qPCR results showing direct binding of SALL1 to the DNA promoter region of *MGST1* (n = 3). (h) Luciferase reporter assay results showing that SALL1 increases *MGST1* promoter reporter activity (n = 6). (i) CCK8 results showing that *MGST1* overexpression rescues the proliferation inhibition of RB cells after *SALL1* knockdown, and knockdown of *MGST1* reduces the enhanced proliferative capacity of RB cells following *SALL1* overexpression (n = 6). (j-k) Colony formation assay results showing that *MGST1* overexpression rescues the proliferation inhibition of RB cells after *SALL1* knockdown (n = 3). si-*SALL1* vs si-*SALL1* + OE-*MGST1*, p = 0.007. The statistical analysis for ChIP-qPCR is Student’s t-test, while one-way ANOVA with Tukey’s multiple comparisons test was used for the other statistical analyses. *P < 0.05, **P < 0.01, ***P < 0.001, ns, not significant. Error bars represent mean ± SD.

To elucidate the functional relationship between SALL1 and *MGST1* in RB, this study first established *SALL1* knockdown and overexpression cell models in Y79 cells. qPCR and Western blot results showed that *SALL1* knockdown significantly reduced both mRNA and protein levels of SALL1 and MGST1 (Fig 4a-c). Conversely, *SALL1* overexpression markedly upregulated the transcription and translation of both SALL1 and MGST1 (Fig 4d-f). These findings indicate that SALL1 positively regulates MGST1 expression. To investigate whether this regulation occurs at the transcriptional level through promoter binding, we performed ChIP-qPCR experiments. The results showed that SALL1 binds to the promoter region of *MGST1* (Fig 4g). Subsequently, luciferase reporter gene experiments confirmed that SALL1 significantly enhances the transcriptional activity of the *MGST1* promoter (Fig 4h), further supporting the conclusion that SALL1, as a transcription factor, directly activates *MGST1* expression. Functionally, CCK8 assays revealed that *SALL1* knockdown significantly inhibited the proliferation of Y79 cells. However, overexpression of *MGST1* in this context partially restored the proliferation inhibition phenotype caused by *SALL1* knockdown. Conversely, *SALL1* overexpression increased cellular proliferation, and this enhanced proliferative capacity could be attenuated by concomitant *MGST1* knockdown. (Fig 4i). Similarly, colony formation assays showed that *MGST1* overexpression reversed the decline in colony-forming ability induced by *SALL1* knockdown (Fig 4j-k).These results suggest that MGST1 is a key effector molecule downstream of SALL1 that promotes proliferation. SALL1 may enhance the proliferative activity of RB cells by positively regulating MGST1 expression.

### 3.5 Targeting the SALL1-MGST1 axis in RB inhibits ferroptosis and reverses tumor drug resistance

**Fig 5:**
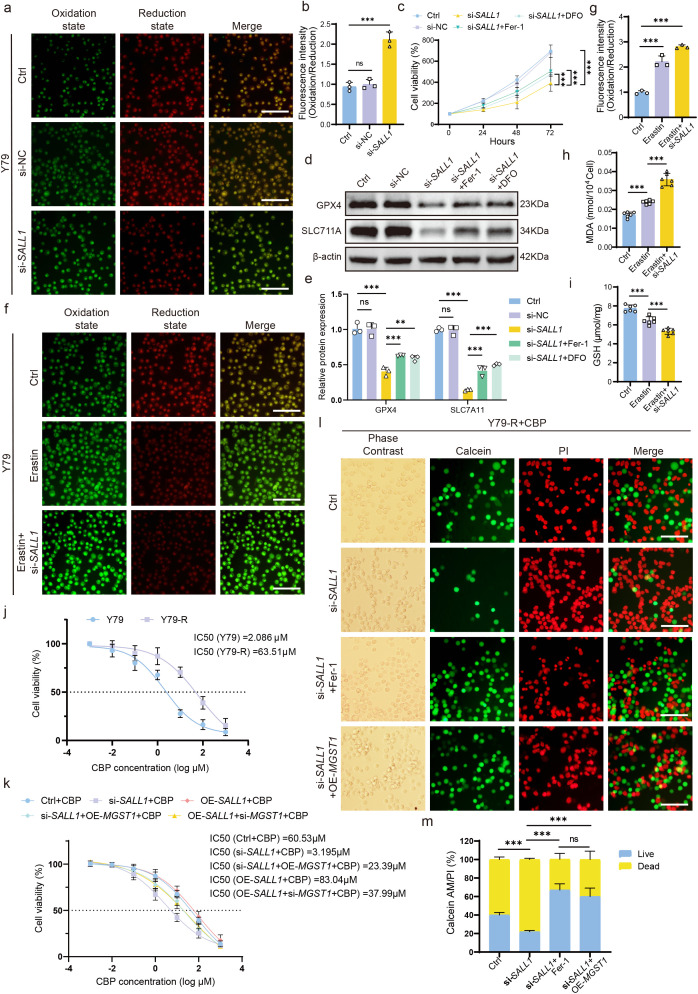
Targeting the SALL1-MGST1 Axis in RB Inhibits Ferroptosis and Reverses Tumor Drug Resistance. (a-b) Lipid peroxidation assay results showing that *SALL1* knockdown increases lipid peroxidation levels in Y79 cells. Scale bar = 100μm, n = 3. (c) CCK8 results showing that ferroptosis inhibition alleviates the proliferation inhibition of Y79 cells caused by *SALL1* knockdown, while overexpression of *SALL1* increases the proliferative capacity of RB cells (n = 6). (d-e) Western blot analysis demonstrated that knockdown of *SALL1* decreased the expression of ferroptosis-related proteins, while the addition of Fer-1 and DFO increased their expression (n = 3). GPX4, si-*SALL1* vs si-*SALL1* + DFO, p = 0.004. (f-g) Lipid peroxidation assay results showing that the ferroptosis inducer increases lipid peroxidation levels in Y79 cells after *SALL1* knockdown. Scale bar = 100μm, n = 3.(h) MDA assay results showing that the ferroptosis inducer increases lipid peroxidation levels in Y79 cells after *SALL1* knockdown (n = 6). (i) GSH assay results showing that the ferroptosis inducer decreases the antioxidant capacity of Y79 cells after *SALL1* knockdown (n = 6). (j) IC50 results showing stronger resistance in Y79-R cells (n = 6). (k) IC50 results showing decreased resistance in Y79-R cells after *SALL1* knockdown, whereas overexpression of *SALL1* enhanced it. (n = 6). (l-m) Live/dead cell double-staining assay results showing higher sensitivity of Y79-R cells to CBP after *SALL1* knockdown. Scale bar = 100μm, n = 3. Statistical analysis is one-way ANOVA with Tukey’s multiple comparisons test. *P < 0.05, **P < 0.01, ***P < 0.001. Error bars represent mean ± SD.

To further investigate the functional role of the SALL1-MGST1 axis in RB, we first examined the effect of *SALL1* knockdown on lipid peroxidation levels in Y79 cells. Lipid peroxidation assays revealed that *SALL1* knockdown significantly increased lipid peroxidation levels in Y79 cells (Fig 5a-b), suggesting enhanced sensitivity to ferroptosis. To further validate the correlation of this phenotype with ferroptosis, we treated the cells with the ferroptosis inhibitor Ferrostatin-1 (Fer-1) and deferoxamine (DFO). CCK-8 assay results showed that treatment with Fer-1 and DFO significantly rescued the proliferation inhibition of Y79 cells caused by *SALL1* knockdown (Fig 5c). We further examined ferroptosis-related proteins in Y79 cells by Western blot. The results showed that the expression of both GPX4 and SLC7A11 decreased following SALL1 knockdown, while their expression increased after treatment with Fer-1 and DFO (Fig 5d-e). These results indicate that the proliferation defect induced by *SALL1* knockdown is partially dependent on the ferroptosis pathway. Subsequently, we used the ferroptosis inducer Erastin to further intervene in the cells. Erastin treatment alone significantly induced lipid peroxidation in Y79 cells, while combined treatment with *SALL1* knockdown further elevated lipid peroxidation levels, showing a synergistic effect (Fig 5f-g). To quantitatively assess the extent of oxidative damage, we measured intracellular MDA content and GSH levels (Fig 5h-i). MDA, a stable end product of lipid peroxidation, reflects the degree of cellular oxidative damage, while GSH, a key intracellular antioxidant molecule, indicates the depletion of the antioxidant defense system. The experimental results showed that in the group with *SALL1* knockdown combined with Erastin treatment, MDA content significantly increased, while GSH levels markedly decreased, further confirming a significant enhancement in oxidative stress levels and a decline in antioxidant capacity under these conditions.

To investigate the role of the SALL1-MGST1 axis in chemotherapy resistance, we compared the drug sensitivity of Y79 cells and their CBP-resistant strain, Y79-R. IC50 determination results showed that the IC50 value of Y79-R cells for CBP was significantly higher than that of Y79 cells, confirming their resistant phenotype (Fig 5j). Subsequently, *SALL1* knockdown in Y79-R cells significantly reduced their IC50 value, indicating partial reversal of resistance. This effect could be rescued by *MGST1* overexpression. Conversely, *SALL1* overexpression increased the IC50 values of Y79-R cells, an effect attenuated by *MGST1* knockdown (Fig 5k). Taken together, these results suggest that SALL1-mediated regulation of drug resistance may depend on MGST1. Finally, we assessed cell mortality using a live/dead cell double-staining assay. In CBP-treated Y79-R cells, *SALL1* knockdown significantly increased cell death, while concurrent treatment with Fer-1 or *MGST1* overexpression markedly alleviated this death phenotype (Fig 5l-m). These results consistently indicate that SALL1 inhibits ferroptosis by upregulating MGST1 expression, thereby enhancing the resistance of RB cells to CBP.

### 3.6. Schematic diagram of the mechanism by which targeting the SALL1-MGST1 axis in RB inhibits ferroptosis and reverses tumor drug resistance

**Fig 6:**
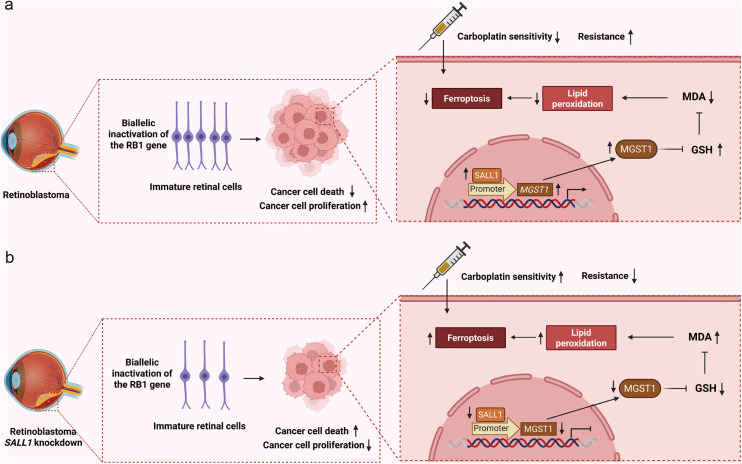
Schematic Diagram of the Mechanism by Which Targeting the SALL1-MGST1 Axis in RB Inhibits Ferroptosis and Reverses Tumor Drug Resistance.

As shown in Fig 6, this study revealed the mechanism by which SALL1 regulates CBP sensitivity in RB. SALL1 directly binds to the promoter region of *MGST1* and activates its transcription. The upregulated MGST1 inhibits the accumulation of lipid peroxides, thereby blocking ferroptosis and reducing the sensitivity of tumor cells to CBP while promoting proliferation (Fig 6a). After *SALL1* knockdown, *MGST1* transcription decreases, and its expression declines, leading to increased lipid peroxidation levels and enhanced ferroptosis, ultimately promoting cell apoptosis and significantly improving CBP chemosensitivity (Fig 6b). In summary, the SALL1-MGST1 axis promotes RB proliferation and CBP resistance by inhibiting ferroptosis. This mechanism provides a new perspective for understanding chemotherapy resistance in RB and lays a theoretical foundation for the development of novel therapeutic strategies targeting this pathway.

## 4. Discussion

The diagnosis and treatment of cancer have always been challenging, especially for rare cancers, posing a significant burden on patients, caregivers, and clinicians. In RB, the emergence of chemotherapy resistance, including primary, adaptive, and acquired resistance, remains a major obstacle in current treatment, severely affecting patient prognosis [30]. In this study, we found that the transcription factor SALL1 in RB can directly bind to the promoter region of the *MGST1* gene and activate its transcription. The upregulated expression of MGST1 effectively inhibits the accumulation of intracellular lipid peroxides, thereby blocking the ferroptosis process. This mechanism significantly reduces the chemosensitivity of tumor cells to CBP while promoting cell proliferation. Notably, inhibiting the SALL1-MGST1 signaling axis can effectively restore cellular sensitivity to ferroptosis and suppress tumor growth. This strategy focuses on the regulation of cell death modalities rather than traditional proliferation inhibition, offering a new therapeutic direction for overcoming chemotherapy resistance.

In recent years, multiple studies have gradually revealed the therapeutic potential of non-apoptotic cell death pathways in targeting apoptosis-resistant tumors [31]. Given the significant differences in signal transduction and execution mechanisms among different cell death modalities, it is crucial to thoroughly evaluate the integrity of key components of cell death in specific cancers [32]. Ferroptosis, an iron-dependent novel programmed cell death modality characterized by the accumulation of lipid peroxides, has garnered increasing attention for its role in tumor development, progression, and treatment resistance. In RB, multiple studies have revealed the diversity of ferroptosis regulation: YAP expression inhibition enhances RB sensitivity to cisplatin/etoposide by promoting lipid peroxidation and ferroptosis, accompanied by a shift in mitochondrial metabolism toward fatty acid oxidation dependence [33]. RB1 loss/E2F activation increases cellular sensitivity to ferroptosis by upregulating ACSL4 expression and increasing arachidonic acid phospholipid synthesis [34]. taconate 4-octyl ester induces oxidative damage and enhances ferroptosis sensitivity in drug-resistant RB cells by activating NCOA4-mediated ferritin autophagy, promoting free iron release, and facilitating the Fenton reaction [35]. These findings collectively demonstrate that targeting the ferroptosis pathway can effectively enhance the sensitivity of RB to conventional chemotherapy drugs. Against this backdrop, our study further reveals a novel ferroptosis regulatory pathway: the transcription factor SALL1, as a key upstream regulator, can directly bind to the *MGST1* gene promoter and activate its transcription. MGST1, an important antioxidant enzyme, inhibits the ferroptosis process by scavenging lipid peroxides, thereby promoting tumor cell proliferation and inducing resistance to CBP. This mechanism not only enriches the network system of ferroptosis regulation in RB but also provides a new potential target—the SALL1-MGST1-ferroptosis axis—for overcoming chemotherapy resistance.

The resistance mechanisms of platinum-based chemotherapy drugs are highly complex, involving multiple layers such as autophagy activation, mutations in drug resistance-related genes, and dysregulation of various cell death pathways [36]. The disruption of redox homeostasis is an indispensable factor contributing to chemotherapy resistance in tumor cells. In recent years, the reprogramming of the tumor metabolic microenvironment, particularly the disruption and reconstruction of redox homeostasis, has been recognized as a key link in the formation of drug resistance. Under chemotherapy pressure, tumor cells can establish a new balance in the ROS-antioxidant system through theredox remodeling” process, thereby evading drug-induced killing [37]. The role of MGST1 in tumor drug resistance has been confirmed by multiple studies. In various tumors, high MGST1 expression is associated with poor prognosis and chemotherapy resistance. Research has shown that MGST1 inhibits ferroptosis in cancer cells by binding to ALOX5, thereby reducing lipid peroxidation and leading to poor prognosis in patients with pancreatic ductal adenocarcinoma. Additionally, in gastric cancer, MGST1 has been demonstrated to inhibit ferroptosis by activating AKT and enhancing the Wnt/β-catenin pathway, thereby promoting drug resistance phenotypes and disease progression [38]. These findings collectively establish MGST1 as an important potential target for reversing drug resistance. Our study further expands the understanding of MGST1’s mechanism in RB drug resistance. Our experimental results show that *SALL1* knockdown effectively inhibits MGST1 expression, thereby inducing lipid peroxidation and ferroptosis, significantly suppressing RB cell proliferation, and enhancing their sensitivity to CBP. This discovery not only reveals the key role of the SALL1-MGST1 axis in ferroptosis regulation in RB but also provides a new targeted strategy for overcoming platinum-based drug resistance.

This study has several limitations. First, all experiments were conducted using cell line models. While they have revealed, to a certain extent, the role of the SALL1-MGST1 axis in regulating ferroptosis and CBP resistance, the lack of *in vivo* experimental validation means that its physiological and pathological relevance and translational value still require further assessment. Second, the specific co-factors and epigenetic modification mechanisms involved in SALL1’s regulation of *MGST1* transcription remain unelucidated, and how upstream signals influence this pathway also requires further investigation. Third, while our immune microenvironment analysis using CIBERSORT provided insights into immune cell infiltration, this algorithm estimates relative immune cell fractions and does not incorporate explicit tumor purity adjustments. The inferred immune proportions could therefore be influenced by varying tumor cellularity across samples, and future studies including tumor purity estimates or sensitivity analyses would enhance the robustness of these findings. Additionally, our current focus has been primarily on CBP resistance, and whether this pathway is involved in RB resistance to other chemotherapy drugs remains unknown, necessitating systematic research. Furthermore, although targeting either SALL1 (a transcription factor) or MGST1 (an enzyme) holds potential for reversing chemoresistance, their translational prospects differ. Targeting SALL1, a transcription factor, may pose challenges in selectivity and off-target effects with small-molecule inhibitors or gene-editing approaches, whereas MGST1, as an enzymatic target, might be more amenable to inhibitor design. Future studies should assess the potential retinal toxicity of targeting this axis, particularly on developing or mature retinal function.

In summary, this study is the first to reveal a novel mechanism by which SALL1 inhibits ferroptosis and promotes RB proliferation and CBP resistance through transcriptional activation of MGST1 expression. These findings not only deepen our understanding of RB resistance mechanisms but also provide a potential new target—the SALL1-MGST1 signaling axis—for reversing chemotherapy resistance. Subsequent studies need to validate the function of this pathway in models closer to physiological conditions and explore feasible strategies for its clinical application in drug resistance prevention and treatment.

## Supporting information

S1 Table Primer sequences used in this study. The primer sequences used for qRT-PCR analysis are listed in Supplementary Table 1.
(XLSX)

S2 Table Primer sequences used for ChIP-qPCR analysis. The primer sequences used for ChIP-qPCR validation are listed in Supplementary Table 2.
(XLSX)

S3 Table Complete statistical data for SALL1 differential expression analysis. The complete statistical results for SALL1, including log₂FC values, P values, and FDR values, are provided in Supplementary Table 3.
(XLSX)

S4 Table Detailed sample-level statistical parameters for immune cell infiltration analysis. The detailed sample-specific P values and root mean square error (RMSE) values for each immune cell type are provided in Supplementary Table 4.
(XLSX)

S5 Table Dose-response curve fitting parameters and statistical details. The key parameters of the fitted dose-response curves, including the Hill slope and its 95% confidence intervals (95% CIs), are provided in Supplementary Table 5.
(XLSX)

S1 Fig Raw images. (PDF)

## Abbreviation:

RB: Retinoblastoma
CBP: Carboplatin
SALL1: Spalt-Like Transcription Factor 1
MGST1: Microsomal glutathione S-transferase 1
ATCC: American Type Culture Collection
GEO: Gene Expression Omnibus
DEGs: Differentially Expressed Genes
GO: Gene Ontology
KEGG: Kyoto Encyclopedia of Genes and Genomes
ES: Enrichment Scores; fgsea, Fast Gene Set Enrichment Analysis
FDR: False Discovery Rate
CCK8: Cell Counting Kit-8
MDA: Malondialdehyde
GSH: Glutathione
BP: Biological Processes
CC: Cellular Components
MF: Molecular Functions
Treg s: Regulatory T Cells.
