# Supplementary material for: The role of SALL1-MGST1 axis-mediated ferroptosis inhibition in chemoresistance of retinoblastoma
Source: PLoS One. 2026 Jul 6;21(7):e0353000. doi: 10.1371/journal.pone.0353000 (PMC13336194; doi:10.1371/journal.pone.0353000)
Supplement: S1 Fig — (PDF) [file pone.0353000.s006.pdf]

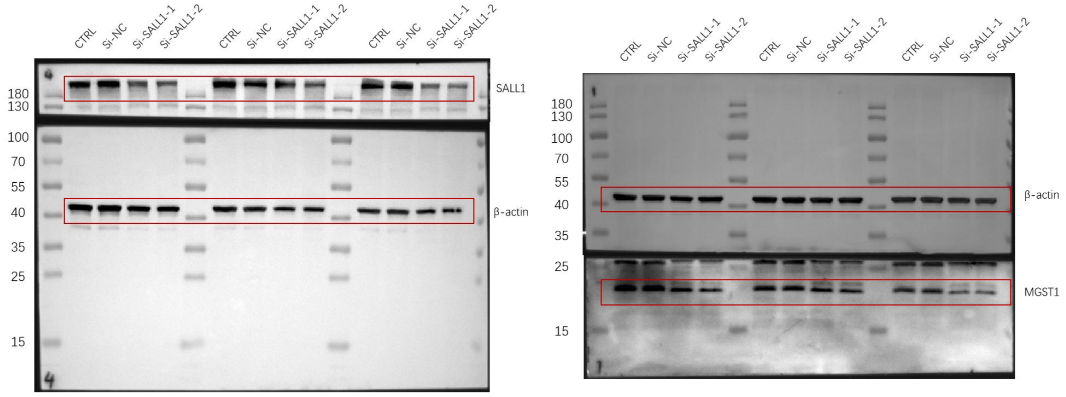

Supplementary Figure 1. Presentation of Full Membrane Results from Western Blot Experiments. Corresponds to Figure 4 b-c in the original text.

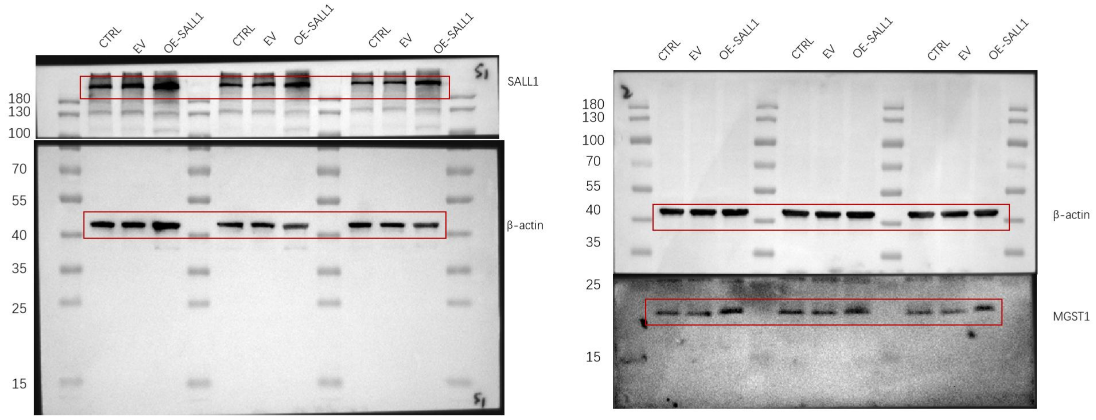

Supplementary Figure 2. Presentation of Full Membrane Results from Western Blot Experiments. Corresponds to Figure 4 e-f in the original text.

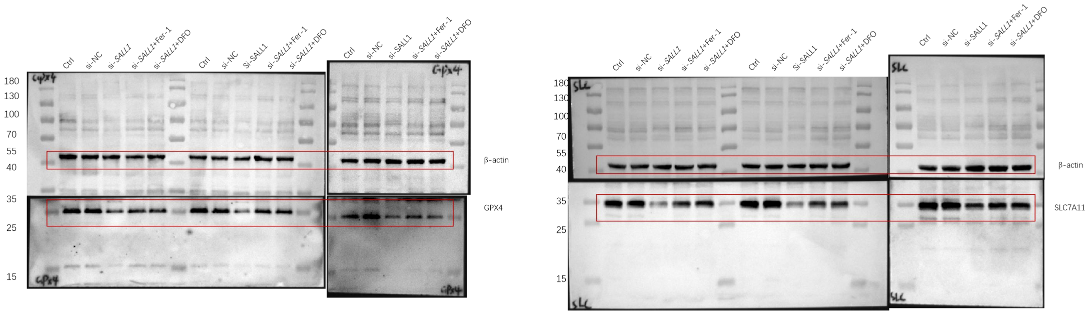

Supplementary Figure 3. Presentation of Full Membrane Results from Western Blot Experiments. Corresponds to Figure 5 e-f in the original text.
